# Examining the Effectiveness of Educational Tools and Elements Used in Medical Textbooks: Students' Perspective

**DOI:** 10.7759/cureus.59264

**Published:** 2024-04-29

**Authors:** Ahmed Eldeib, Omar Eldeib, Ayman Mohammed

**Affiliations:** 1 Department of Surgery, State University of New York (SUNY) Downstate Health Sciences University College of Medicine, Brooklyn, USA; 2 Department of Medical Education, Alfaisal University College of Medicine, Riyadh, SAU

**Keywords:** educational resources, medical education, educational outcomes, educational tools, medical textbook

## Abstract

Background

As textbooks constitute a foundational component of medical education, it is imperative to conduct a rigorous evaluation of their efficacy by examining the fundamental elements and tools utilized within these textbooks and how multiple factors may alter the effectiveness of such components.

Materials and methods

We conducted a cross-sectional, survey-based study where 251 subjects anonymously rated the effectiveness of different tools and elements/factors used in medical textbooks using 5-point Likert scales. The population surveyed included year 1 to 5 medical students at Alfaisal University, where students are admitted to medical school directly after high school, with an approximately equal male-to-female admission rate. Students were asked to rate the effectiveness of the following tools: chapter summary, end-of-chapter questions, tables of contents, graphic elements, diagrams, tables, flowcharts, mind maps, sidebars, and links to online resources. In admission, they were asked to rate textbooks regarding the following elements/factors: text clarity, directness to the concept intended, flow of ideas, language complexity, and clinical (vertical) and multidisciplinary (horizontal) integration. The Chi-square, post-hoc, Spearman’s correlation, and Kruskall-Wallis tests were used in the statistical analysis to determine the differences and correlations in the students’ self-ratings of different variables.

Results

The students rated tools such as diagrams and graphic elements as the most effective. Graphic elements were rated as significantly more effective by first- and fourth-year students. This corresponds to being exposed to new environments (the first exposure to the study of medicine and the first exposure to clinical clerkship), indicating their effectiveness in introducing new concepts and setting of change. Furthermore, end-of-chapter summaries and questions were rated significantly as being more effective by females (P<0.05). The aim behind using textbooks and the frequency might be governed by how students rate textbooks regarding multiple elements or factors, including directness to the concept intended, flow of ideas, and language complexity. Interestingly, all the above findings were consistent throughout all grade point average groups.

Discussion

In the rising age of e-learning, our study shows that the tools employed by textbooks remain effective. Our findings are partially consistent with existing literature, which underscores correlations between demographic variables and learning styles. We show that the effectiveness of various tools employed in textbooks can vary depending on the educational setting. While demographic factors generally did not impact students' perceptions of tool effectiveness, gender-specific differences were observed in the perceived effectiveness of end-of-chapter summaries and questions, with female students rating them as more effective, which aligns with existing literature.

Conclusions

Our study shows that students still perceive the tools employed by textbooks as effective. The perceived effectiveness of various tools utilized within textbooks may be influenced by certain demographic factors and settings, with graphics often proving the most efficacious. This study provides valuable insights for authors of medical textbooks, helping them optimize the usage of various tools by considering the specific characteristics and requirements of the intended audience.

## Introduction

In the modern landscape characterized by rapid technological advancements, there appears to be a notable trend among medical students toward relying on alternative sources, particularly online platforms, for accessing information beyond traditional textbooks [1-3]. Although this might be taken lightly, the fact that students are neglecting medical textbooks is worrying due to the potential disadvantages electronic resources might have [4-7]. The large repository of easily accessible information might result in reduced emphasis on memorization and comprehension of fundamental concepts, resulting in superficial learning [4]. Moreover, such electronic resources might reduce exposure to collateral, potentially useful information that might aid a full understanding of concepts [4]. Other disadvantages include the reliability of some online sources, lack of professionalism, and negative perceptions from patients and other healthcare workers, as non-medical professionals might have access to the same information source as their providers [4-7].

Various factors have been postulated to potentially impact the utilization patterns of medical students regarding textbooks, including text clarity, language complexity, directness to the concept intended, flow of ideas, clinical/multidisciplinary integration, and effectiveness of the different aiding tools used by textbooks [8-10]. It is reasonable to assume that text clarity and language complexity play an important role, as complex, unclear text may hinder comprehension, thus pushing students to alternative sources [8]. In the literature, medical textbooks have been shown to be of high complexity, requiring a high educational level for comprehensive understanding [8].

Additionally, factors like a streamlined flow of ideas and directness in conveying concepts are crucial, as a disjointed structure may hinder understanding and diminish the utility of acquired knowledge, further reinforcing the perception of textbooks as ineffective. Given the vast amounts of information that must be absorbed by medical students within time constraints, the importance of directness in conveying concepts is highlighted. In fact, one of the most important advantages of electronic sources in comparison to paper-based textbooks is searchability, allowing students to reach the required information in less time and with less effort [11].

Multidisciplinary (horizontal) integration is proving very adventitious in medical education, as knowledge acquired in an integrative manner is more likely to be more applicable and less likely to contain unnecessary, out-of-context information [12]. This is highlighted by the widespread appreciation of horizontal integration among medical students globally [12,13]. Moreover, the integration of basic and clinical knowledge (vertical integration) has proven to have profound benefits in helping students deeply comprehend, retain, and apply knowledge [13,14]. Because there is an increasing worldwide trend toward supporting vertical integration in the field of medical education [15-19], we believe that medical textbooks providing such integration will be valued by both students and teachers [13].

The majority of medical textbooks nowadays utilize aiding tools like chapter summaries, end-of-chapter practice questions, sidebars, links to online resources, and visual tools [20-22]. The extensive array of tools utilized by textbooks is essential in facilitating comprehension and robust transfer of information to the intended audience [20-26], which is highlighted by the fact that medical students don’t only prefer one aiding tool; the majority of the students prefer the use of multiple forms of aiding tools [27,28]. Moreover, as medical students exhibit diversity in their learning styles [20-23], an array of different tools should be used to ensure that the majority of students are satisfied [24]. This can, in turn, be associated with student performance, as student motivation and performance are improved when the teaching method is adapted to the student’s learning style [24,25].

For instance, diagrams, serving as a graphical representation of written text, and flowcharts, depicting the mechanisms of a series of events, may prove to be of great benefit to visual learners [24]. On the other hand, tables and end-of-chapter summaries and questions might be more useful for read/write learners [24]. Other effective but less frequently used visual tools include mind maps or concept maps, which are effective ways of showing links between a main concept or topic and many subconcepts and subtopics [26]. A study conducted by Nesbit and Adesope involving 5818 students across different academic levels found that students perceived concept mapping to be more effective than simply reading text passages [26].

Lastly, modern tools such as links to online resources such as videos and animations might prove to be a great benefit to students who are auditory or kinesthetic learners [24]. With the diminishing use of textbooks among students [1-3], it becomes crucial to assess whether the tools utilized by textbooks are still perceived as effective. This evaluation is imperative as it may aid in revealing the reasons behind the reduction in utilization. We aim to explore which tools students think are most effective and link the effectiveness of the different tools to potential factors that might modify or relate to that effectiveness, like the previous educational system, gender, and grade point average (GPA). Moreover, because students use textbooks with different frequencies and for different purposes (as primary sources, reliable references, or aiding tools to help them understand unclear concepts), it is crucial to assess whether the effectiveness of these tools varies with all of the above.

## Materials and methods

This study was designed to be a cross-sectional, survey-based study where the subjects would autonomously and privately assess their views toward the items in the questionnaire. The study targeted Alfaisal University medical students who had just completed at least one year of medical school. No institutional research board approval is required per internal policy. This study is in compliance with the Declaration of Helsinki developed by the World Medical Association (WMA, 2000), which specifies the ethical principles when human subjects are involved.

Population and procedure

The questionnaire was distributed through the university-issued email to all registered medical students who just finished years 1-5 at Alfaisal University, Saudi Arabia. The surveyed population included all students currently in the basic (years 1 and 2), preclinical (year 3), clinical (years 4-5), and intern components.

Moreover, to ensure a higher response number, several reminders were delivered to the students via their official Alfaisal email and social networks. Furthermore, to ensure a representative sample was obtained, supplementary hard copies were provided to the population with lacking responses.

Questionnaire

The students’s responses were collected using a questionnaire, which was specifically designed by the team after an extensive review of the related literature and aimed at obtaining sufficient demographic factors, determining the subjects' behavior (as a primary source, reference, or just to clarify unclear concepts), and frequency of using textbooks, along with the aim behind usage. Moreover, we aimed to assess students’ opinions regarding directness to the concept intended, flow of ideas, text clarity, language complexity, and multidisciplinary (horizontal) and clinical (vertical) integration. Furthermore, we aimed to assess the students' perceived effectiveness of different tools employed by textbooks.

The questionnaire begins by surveying demographic data like gender, year, GPA, and previous education system, along with several questions aiming at subjectively determining the frequency of usage of textbooks and how students use their textbooks if they use textbooks at all.

The questionnaire assesses the effectiveness of the current tools and different elements by utilizing a 5-point Likert scale, with 5 being the most effective and 1 being the least effective.

Statistical analysis

The data were initially processed to analyze the demographic characteristics of the study subjects and the frequency distribution of the measured items in the tool and element domains. First, the Kruskall-Wallis test was used to determine differences in self-ratings among students with different academic years, academic performances measured by GPA, and previous high school systems. The Wilcoxon rank-sum test was utilized to determine gender-specific effects on items in both domains. Next, the Chi-square test was performed to determine the association between demographics and the preferred format of the textbook. Finally, Spearman's correlation was used to assess the significance and strength of the correlation between the tested items. The data analysis was conducted using SPSS Statistics version 20 (IBM Corp. Released 2011. IBM SPSS Statistics for Windows, Version 20.0. Armonk, NY: IBM Corp.). The values are represented as the mean ± SD.

## Results

Demographic characteristics of the students

A total of 251 medical students from all different academic years of medicine at Alfaisal University responded to the survey. Of the respondents, 57% (N=144) were male and 42% (N=107) were female. The majority of students were from the first year (36%, N=91), while the lowest number was from the fifth year (3%, N=8). Most of the students had a GPA of more than 3.5 (55%, N=138), while a smaller number of students (11%, N=28) had a GPA lower than 3. With regard to the previous educational system, most of the students completed Saudi high school before joining college (53%, N=132). In terms of using medical textbooks, most of the students have utilized medical textbooks as an aiding source (37%, N=93), while a lower number of students (19%, N=48) have declared that textbook is the primary tool of the study. The complete demographic characteristics of the study subjects are depicted in Table 1.

**Table 1:** Demographic characteristics of study subjects GPA: grade point average

Variables	N (%)	
Gender		
Male	144 (57%)	
Female	107 (42%)	
Academic year		
First year	91 (36%)	
Second year	70 (27%)	
Third year	58 (23%)	
Fourth year	24 (10%)	
Fifth year	8 (3%)	
Academic performance (GPA)		
Below 3		28 (11%)
3.0-3.49		85 (34%)
3.5-4.0		138 (55%)
Previous educational system		
Saudi		132 (53%)
British		52 (21%)
American		53 (21%)
Others		14 (6%)
Purpose of using a medical textbook		
As a primary		48 (19%)
As a reference		76 (30%)
As aiding source		93 (37%)

Students’ self-ratings of the tools and elements used in medical textbooks

**Figure 1:**
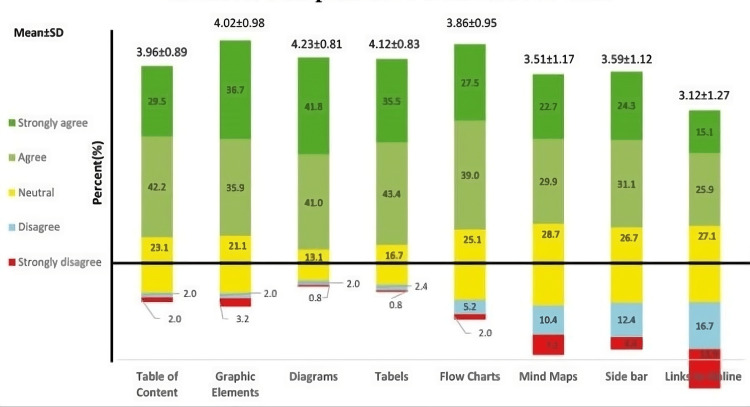
Students’ self-ratings of the various tools employed in textbooks Bars represent the percentage of subjects responding (total N=251). Colors represent various Likert scale responses (dark green = strongly agree, light green = agree, yellow = neutral, blue = disagree, and red = strongly disagree). The mean Likert scale response ±SD is represented above the bar. Statistical significance: P<0.05

Among all the tools assessed in the study, students have rated diagrams as most effective with a mean±SD of 4.23±0.81 and graphic elements with a mean±SD of 4.02±0.98. On the contrary, online links and chapter questions were ranked the lowest, with a mean±SD ranking of 3.12±1.27 and 3.16±1.23, respectively. This is depicted in Figure 1.

Effects of demographic factors on the tools used in medical textbooks

Gender

**Figure 2:**
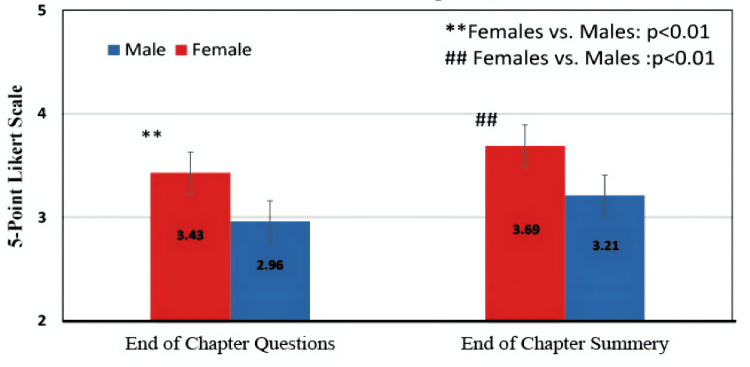
The effect of gender on the perceived effectiveness of chapter summaries and end-of-chapter questions Bar colors represent gender (red = female, blue = male). Bars represent the mean Likert scale response ±SD. Statistical significance: P<0.05

Female students tend to rate chapter summaries and end-of-chapter questions as more effective in comparison to their male peers, with P-values of 0.0029 and 0.0027, respectively. This is depicted in Figure 2.

Other than that, all other tools were rated similarly between the two genders, including tables of contents, graphic elements, diagrams, tables, flowcharts, mind maps, sidebars, and links to online resources, with P-values of 0.715, 0.80, 0.84, 0.59, 0.77, 0.34, 0.83, and 0.41, respectively.

Academic Year

Chapter summary: First-, second-, third-, and fourth-year students rated end-of-chapter summaries more effective when compared to fifth-year medical students, with P-values 0.017, 0.007, 0.045, and 0.027, respectively. Moreover, fourth-year students utilize chapter summaries more frequently than second-year students, with a P-value of 0.011.

Chapter questions: First-, second-, third-, and fourth-year students rated chapter questions more effective compared to fifth-year medical students, with P-values 0.016, 0.04, 0.006, and 0.004, respectively.

**Figure 3:**
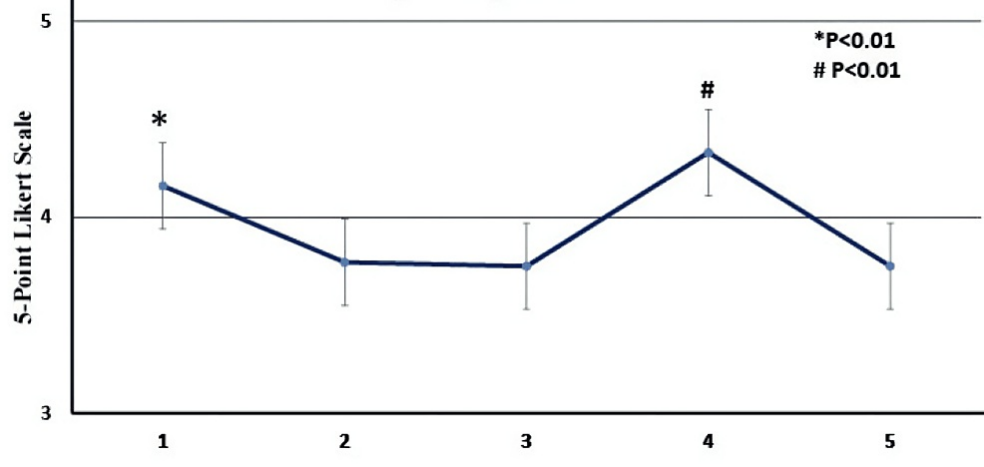
Rating of graphic elements in the different academic years The X-axis represents the academic year. Bars represent the mean Likert scale response ±SD. Statistical significance: P<0.05

Graphic elements: First- and fourth-year students tend to use graphic elements more than the second year, with P-values 0.002 and 0.006, respectively. This is depicted in Figure 3.

Links to online resources: Third-year students are more directed toward using online resources compared to their peers from the second, fourth, and fifth years, with P-values of 0.023, 0.018, and 0.015, respectively.

However, the academic year did not influence students’ self-ratings of tables of contents, diagrams, tables, flowcharts, mind maps, and sidebars, with P-values of 0.18, 0.89, 0.19, 0.52, 0.16, and 0.14, respectively.

GPA

Upon comparing the self-evaluation of students with different academic performances, all the tools had similar ratings, including tables of contents, diagrams, tables, flowcharts, mind maps, sidebars, links to online resources, chapter summaries, chapter questions, and graphic elements, with P-values of 0.73, 0.16, 0.14, 0.09, 0.38, 0.77, 0.61, 0.40, 0.896, and 0.06, respectively.

Previous Educational System

Graphic elements: Students admitted from British schools rated graphic elements more effective than those from the American system, with a P-value of 0.005.

Diagrams: Students admitted from British schools demonstrate a higher rating of diagrams than those from the American system, with a P-value of 0.036. However, students from Saudi educational backgrounds utilize diagrams more than those from American schools, with a P-value of 0.008.

Involvement in different educational systems previously did not depict a difference in students’ self-assessment of tables, flowcharts, mind maps, sidebars, and links to online resources, with P-values of 0.195, 0.63, 0.45, 0.72, 0.68, 0.24, 0.86, and 0.27, respectively.

Purpose of Using Medical Textbooks

Tables: Students who use medical textbooks as a reference have a higher rating of tables than those who consider them as aiding tools, with a P-value of 0.005.

The purpose of using a medical textbook did not influence students’ scoring of graphic elements, tables of contents, diagrams, flowcharts, mind maps, sidebars, links to online resources, chapter summaries, and chapter questions, with P-values of 0.66, 0.55, 0.53, 0.999, 0.71, 0.75, 0.96, 0.40, and 0.056, respectively.

Frequency of Using Medical Textbooks

Tools positively correlated with the frequency of using textbooks are diagrams (P=0.004, r=0.20), graphic elements (P<0.001, r=0.29), and tables (P=0.037, r=0.14).

Effects of demographic factors on rating medical textbooks with regard to specific elements

Gender

There was no gender-specific effect on students’ self-ratings of text clarity, directness to the concept intended, flow of ideas, language complexity, and clinical and multidisciplinary integration, with P-values of 0.41, 0.97, 0.59, 0.997, 0.82, and 0.31, respectively.

Academic Year

Students in all academic years rated medical textbooks similarly with regard to text clarity, directness to the concept intended, flow of ideas, language complexity, and clinical and multidisciplinary integration, with P-values of 0.09, 0.10, 0.15, 0.28, 0.11, and 0.32, respectively.

GPA

Students with different academic performances rated medical textbooks similarly with regard to text clarity, directness to the concept intended, flow of ideas, language complexity, and clinical and multidisciplinary integration, with P-values of 0.97, 0.48, 0.52, 0.68, 0.90, and 0.81, respectively.

Previous Educational System

Students coming from different educational backgrounds have not demonstrated any significant differences in their ratings of text clarity, directness to the concept intended, flow of ideas, language complexity, and clinical and multidisciplinary integration, with P-values of 0.94, 0.77, 0.319, 0.38, 0.98, and 0.75, respectively.

Purpose of Using Medical Textbooks

Directness to the content intended: Students who use textbooks as the primary tool rate textbooks higher with regard to directness to the concept intended compared to those who consider it a reference, with a P-value of 0.014

Flow of ideas: Textbooks are rated as demonstrating a better flow of ideas among those who use the textbook as the primary tool compared to those who utilize it as a reference or as an aiding tool, with P-values of 0.002 and 0.005, respectively.

**Figure 4:**
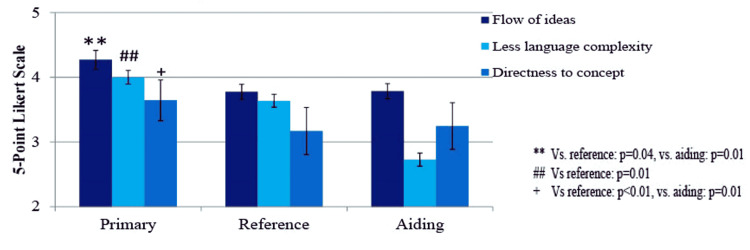
Purpose of using medical textbooks in relation to the rating of different textbook elements Bar colors represent elements (dark blue = flow of ideas, intermediate = directness to the concept intended, light = less language complexity (i.e., simple language). Bars represent the mean Likert scale response ±SD. Statistical significance: P<0.05

Language complexity: Students who utilize medical textbooks as the primary tool perceive them as having more complex language compared to those who use them as a reference or an aiding means, with P-values of 0.044 and 0.005, as depicted in Figure 4.

Frequency of Using Medical Textbooks

**Figure 5:**
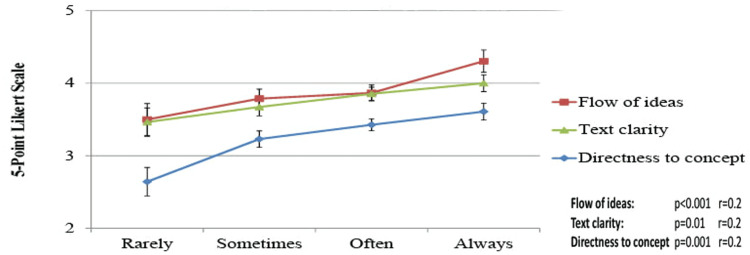
Use of medical textbooks and student ratings of the different elements employed The X-axis represents the frequency of textbook usage. Line colors represent the elements (red = flow of ideas, green = text clarity, and blue = directness to the concept intended). The line statistic is the mean Likert scale response ±SD. Statistical significance: P<0.05

A higher rating of factors, including text clarity, directness to the concept intended, and flow of ideas, is positively correlated with more frequent use of medical textbooks (P=0.01, r=0.18; P=0.001, r=0.23; and P<0.001, r=0.24) and clinical integration (P=0.04, r=0.14). This is depicted in Figure 5.

Associations Between the Demographics and Format Preferred for Medical Textbooks

All the factors, including gender, academic year, GPA, previous educational background, and purpose of using medical textbooks, are not associated with the students’ preference for text formats, with P-values of 0.90, 0.28, 0.24, 0.21, and 0.23, respectively. Moreover, the frequency of using medical textbooks is not related to the format of the textbook preferred by the students (P=0.12).

Correlations Between the Various Tools and Elements Utilized in Medical Textbooks

The associations/correlations between the various tools and elements used in medical textbooks are shown in Table 2.

**Table 2:** Associations/correlations between the various tools and elements NSC: no statistically significant correlation, statistically significant: P<0.05

	Table of content	Text clarity	Directness to the concept intended	Flow of ideas	Language complexity	Chapter summary	Chapter questions	Graphic elements
Diagrams	P<0.00, r=0.26	P<0.001, r=0.33	P=0.007, r=0.17	P<0.001, r=0.34	NSC	P<0.001, r=0.30	P=0.007, r=0.17	P<0.001, r=0.59
Tables	P=0.001, r=0.21	P<0.001, r=0.27	P=0.017, r=0.15	P=0.003, r=0.19	P=0.028, r=0.14	P<0.001, r=0.27	P=0.001, r=0.20	P<0.001, r=0.42
Flowcharts	P=0.004, r=0.18	P<0.001, r=0.28	P<0.001, r=0.22	P=0.002, r=0.20	NSC	P<0.001, r=0.32	P<0.001, r=0.36	P<0.001, r=0.46
Sidebar	P=0.002, r=0.198	P<0.001, r=0.23	P=0.001, r=0.29	P=0.001, r=0.22	NSC	P<0.001, r=0.32	P<0.001, r=0.32	P<0.001, r=0.28
Clinical integration	P=0.001, r=0.21	P=0.001, r=0.215	P<0.001, r=0.32	P<0.001, r=0.35	P=0.03, r=0.14	P<0.001, r=0.26	P<0.001, r=0.28	P<0.001, r=0.32
Multidisciplinary integration	P=0.029, r=0.14	P<0.001, r=0.33	P<0.001, r=0.30	P<0.001, r=0.29	NSC	P<0.001, r=0.28	P<0.001, r=0.31	P<0.001, r=0.29
Mind maps	NSC	NSC	P<0.001, r=0.27	P=0.003, r=0.19	NSC	P<0.001, r=0.34	P<0.001, r=0.36	P<0.001, r=0.25
Links to online resources	NSC	NSC	P=0.012, r=0.16	NSC	NSC	P<0.001, r=0.23	P<0.001, r=0.35	NSC

## Discussion

Our study highlights gender-specific differences with regard to the perceived effectiveness of some of the tools employed by textbooks. Our results show that female students rated chapter summaries and end-of-chapter questions as more effective compared to males, which is consistent with the study conducted by Wehrwein et al. This study concluded that females prefer learning through reading and writing in comparison to males [29]. Chapter summaries and end-of-chapter questions purely involve reading and writing and thus are expected to satisfy the R (reading/writing) learning style classified by Fleming et al. [24]. The fact that different tools are beneficial to different genders raises the question of whether tailored learning has a role to play when it comes to recommending learning resources. Based on our results, we recommend that all book authors try to include more chapter summaries and end-of-chapter questions in their textbooks. This recommendation applies to all books but is especially applicable to textbooks recommended by faculty as primary sources. In view of the above and supported by the fact that it has been shown that student motivation and performance are improved when the teaching tools are adapted to the student’s learning style [24,25], it is crucial for both curriculum planners to supplement any resources they require students to study from and book authors to make sure that they integrate chapter summaries and relevant end-of-chapter questions in their books. This helps reduce any inequality in the way knowledge is presented to different genders and helps provide tailored education to each individual [30]. Given that students who derive no utility from chapter summaries and end-of-chapter questions may opt to disregard them, whereas those who find them beneficial will not be deprived of their advantages. Since all the other tools were rated similarly by both genders, this suggests that there is no differential benefit to these tools with regard to gender.

Moreover, the fact that our students generally rated all tools employed by textbooks positively and that most demographic factors didn’t affect their ratings with regard to tools is a finding in favor of medical textbooks. It shows the consistency and reliability with regard to the tools employed by textbooks.

There are some exceptions to the above, with gender (female students rating summaries/end-of-chapter questions as more effective) and previous educational system (British system students rating graphics/diagrams as more effective). In addition, demographic factors did not have any effects on how students rated textbooks with regard to text clarity, directness to the concept intended, flow of ideas, language complexity, and clinical and multidisciplinary integration, yet again a positive finding, showing no disparities in the perceived effectiveness of textbooks based on demographic factors.

The effectiveness of different tools and elements through the different academic years

The curriculum of Alfaisal University is divided into three phases: phase 1, phase 2, and phase 3. The aims are to understand the normal function of the human body, understand pathophysiology and the interlinked subjects, and understand methods of clinical application of the acquired medical knowledge, respectively. Years 1 and 2 represent phase 1, year 3 represents phase 2, and years 4 and 5 represent phase 3.

Our results showed that students from years 1-4 have a higher rating for chapter summaries and end-of-chapter questions compared to fifth-year medical students. This is very important to discuss, as several studies have concluded that students tend to rely on online sources when it comes to the pure clinical aspects of medicine [1,2,31]. One example would be the study conducted by Cogdill and Moore, which showed that students used MEDLINE most frequently to answer questions that had to do with treatment [31]. Moreover, a study by Prasannan et al. concluded that students use academic databases and Google more frequently than textbooks to find information regarding medication and other clinical aspects [2]. Moreover, another study concluded that only 14% of students use textbooks as a primary source during the final years of medical school, and most use online sources [1]. We speculate that the reason behind fifth-year medical students not preferring chapter summaries and questions is that they don’t require summaries and questions but require detailed information on targeted concepts. For instance, some students may not find reading a summary on hyperthyroidism appealing, but they may express interest in understanding the precise mechanism of action of a drug employed for hyperthyroidism treatment. This explains why they tend to use online resources, as one of their advantages is searchability [11].

Another interesting finding is that first- and fourth-year students tend to rate graphic elements higher than all other years. In the initial year of their medical studies, students encounter the discipline of medicine for the first time, whereas by the fourth year, they undergo a novel experience of transitioning into a hospital setting and becoming acquainted with medical procedures.

Also, third-year medical students rated links to online resources significantly higher than all the other years. This might be due to the fact that it’s their first exposure to pathology and its interrelated subjects such as pharmacology and microbiology, and this might indicate that links to online resources are important whenever students are studying pathology, microbiology, and pharmacology.

The relationship between the perceived effectiveness of the different tools, rating of different elements, and usage behavior

The observation that medical students increasingly disregard medical textbooks and instead rely more frequently on alternative primary sources, particularly online sources [1-3,31], is worrying, as are the disadvantages online sources might pose [4-7]. It proves very necessary to investigate the cause. Our study concludes that students who rate textbooks higher when it comes to the factors/elements (directness to the concept intended and flow of ideas) are both more likely to use textbooks as primary sources and are more likely to use textbooks more frequently. This effect spans all the demographic factors we surveyed. Firstly, when it comes to the directness of the concept intended, one of the principal advantages of electronic sources over paper-based textbooks is their inherent searchability, allowing students to directly reach the required information in less time and with less effort [1,11]. In light of this result and if textbooks are to keep up with the current online sources, making textbooks more direct to the concept intended should be one of the main aims of future book authors.

Secondly, the fact that the flow of ideas determines if students use the textbook as a primary source or not and the frequency of usage is very logical. If students perceive the textbook as having a poor flow of ideas, they will most probably not use it more frequently and not as a primary source. For proper learning to occur, information should be properly structured [32].

Thirdly, our results showed that language complexity is an important factor that determines both the frequency of usage and the aim behind the usage (using books as the primary source). We showed text clarity to be a factor in determining the frequency of usage only. It is worth mentioning that most of our students are not native English speakers. Our results are supported by the multiple reports in the literature concluding that even for native English speakers, a high level of education is required in order for people to understand medical textbooks [8-10]. Moreover, other reports in the literature conclude that ethnic minorities who are non-native English speakers can find great difficulty in reading and interpreting general medical texts [9,33,34].

Clinical (vertical) integration demonstrated a correlation with the frequency of textbook usage among students. We believe this association can be attributed to several factors. Clinical correlates are examined at Alfaisal University right from the outset, motivating students to engage with textbooks that aid in their exam preparation. In addition, we have shown that the more the book archives clinical (vertical) integration, the more frequently it is used by students. This is supported by reports in the literature that conclude that vertical integration has proven beneficial in helping students deeply understand, retain, and apply knowledge [15-19].

Multidisciplinary (horizontal) integration demonstrated no correlation with the frequency of textbook usage among students. This observation may initially seem contradictory, given the widespread recognition of the utility of multidisciplinary integration among students globally [12,13].

We believe this can be explained by the integrated nature of the system at Alfaisal University, where lectures spanning various subjects are delivered in an integrated fashion. Consequently, this fulfills students' requirements for horizontal integration.

It's noteworthy that among the tools examined, only tables were associated with the underlying purpose of usage. Specifically, students who utilize books as references rated tables more favorably compared to those who use books as merely aiding tools. This observation makes logical sense. Tables serve to condense key points, facilitate quick reference, and assist students in accurately obtaining the information needed for activities such as problem-based learning. Conversely, if a student struggles with comprehending a concept, they may not rate tables highly, as tables present information without providing explanations.

It is noteworthy to mention that this study has limitations. This study was predominantly a cross-sectional survey-based study, with the intrinsic limitations of such a study being the subjectivity of responses and nonresponder selection bias. Moreover, the number of fifth-year medical student respondents was limited in comparison to other years (3%, N=8); these results involving fifth-year medical students should be interpreted with caution. Although our study design was aimed at answering specific questions with regard to textbook utilization and the perceived effectiveness of tools employed, the integration of personal interviews into future study designs is likely to be of importance in exploring student perceptions in a more open-ended fashion.

## Conclusions

Our findings suggest that the efficacy of various tools utilized by textbooks may vary depending on the setting. In general, students rated all tools employed by textbooks positively, with graphics and diagrams being rated the highest. Demographic factors generally had no effect on the students' rating of the effectiveness of all tools employed by textbooks or how students rated textbooks with regard to text clarity, directness to the concept intended, flow of ideas, language complexity, and clinical and multidisciplinary integration. The exception to that was gender-specific differences with regard to the perceived effectiveness of end-of-chapter summaries and questions, with those being rated as more effective by female students in comparison to male students, a finding that is consistent with different reports in the literature.

Moreover, we have shown that students tend to rate graphics and diagrams higher compared to their peers when they are initially introduced to medical teaching (first year) as well as when they are first introduced to the clinical setting (fourth year). We have also shown that links to online resources are perceived as more effective upon transitioning from medical disciplines taught in the first and second years to pathology and its interlinked subjects. The aim behind using textbooks and the frequency might be governed by how students rate textbooks with regard to multiple elements/factors, including directness to the concept intended, flow of ideas, and language complexity.

## Appendices

**Table 3:** Questionnaire GPA: grade point average, TOEFL: Test of English as a Foreign Language, IELTS: International English Language Testing System, IGCSE: International General Certificate of Secondary Education

Questionnaire item	Response
Gender	Text
Academic year (the one you just finished)	1/2/3/4/5
GPA	Free numeric
Your score in standardized English exams: (TOEFL)	Free numeric
Your score in standardized English exams: (IELTS)	Free numeric
Your score in standardized English exams: (IGCSE English)	Text
Your previous educational system	Saudi/British/American
Do you use your medical textbooks in studying (even if for one subject/discipline only)?	Yes/no
If yes, then how often do you use them?	Always/often/sometimes/rarely
What best describes your use of them?	As a reference/as a primary source/as an aiding tool
Referring to my preferred or selected medical textbook helps me improve each of the following: (knowledge (recalling data or information))	Strongly agree/agree/neutral/disagree/strongly disagree
Referring to my preferred or selected medical textbook helps me improve each of the following: (comprehension (it helps me demonstrate my understanding))	Strongly agree/agree/neutral/disagree/strongly disagree
Referring to my preferred or selected medical textbook helps me improve each of the following: (application (applying what was learned to clinical context))	Strongly agree/agree/neutral/disagree/strongly disagree
Referring to my preferred or selected medical textbook helps me improve each of the following: (analysis (separating material into component parts and showing relationship between parts))	Strongly agree/agree/neutral/disagree/strongly disagree
Referring to my preferred or selected medical textbook helps me improve each of the following: (organizing (organizing values into priorities, with an emphasis on comparing, relating, and synthesizing))	Strongly agree/agree/neutral/disagree/strongly disagree
Referring to a preferred or selected medical textbook helps me improve each of the following: (valuing (attaching worth to a particular object, phenomenon, or behavior))	Strongly agree/agree/neutral/disagree/strongly disagree
Referring to preferred or selected clinical textbooks helps me improve each of the following: (for students in clinical years only) (responding (attending and reacting to a particular phenomenon))	Strongly agree/agree/neutral/disagree/strongly disagree
Referring to preferred or selected clinical textbooks helps me improve each of the following: (for students in clinical years only) (perception (using sensory cues to guide motor activity))	Strongly agree/agree/neutral/disagree/strongly disagree
Referring to preferred or selected clinical textbooks helps me improve each of the following: (for students in clinical years only) (set (possessing a mental, physical, or emotional state underpinning a readiness to act))	Strongly agree/agree/neutral/disagree/strongly disagree
Referring to preferred or selected clinical textbooks helps me improve each of the following: (for students in clinical years only) (guided response (imitating, following instruction, trial and error))	Strongly agree/agree/neutral/disagree/strongly disagree
Referring to preferred or selected clinical textbooks helps me improve each of the following: (for students in clinical years only) (mechanism (applying learned responses habitually and with increasing confidence))	Strongly agree/agree/neutral/disagree/strongly disagree
Referring to preferred or selected clinical textbooks helps me improve each of the following: (for students in clinical years only) (complex overt response (performing without hesitation or automatically))	Strongly agree/agree/neutral/disagree/strongly disagree
Referring to preferred or selected clinical textbooks helps me improve each of the following: (for students in clinical years only) (adaptation (modifying skills to fit special requirements))	Strongly agree/agree/neutral/disagree/strongly disagree
Referring to preferred or selected clinical textbooks helps me improve each of the following: (for students in clinical years only) (origination (showing creativity based on highly developed skills))	Strongly agree/agree/neutral/disagree/strongly disagree
Rate the following elements/aspects in selected or preferred medical textbooks: (1 = poor, 5 = excellent) (tables of contents (is clear and shows the relationship between and within topics/subtopics))	1-5 Likert scale
Rate the following elements/aspects in selected or preferred medical textbooks: (1 = poor, 5 = excellent) (text clarity (content is understandable from the first read))	1-5 Likert scale
Rate the following elements/aspects in selected or preferred medical textbooks: (1 = poor, 5 = excellent) (direct to concept intended (doesn't contain a lot of unnecessary "filling" sentences))	1-5 Likert scale
Rate the following elements/aspects in selected or preferred medical textbooks: (1 = poor, 5 = excellent) (flow of ideas (is clear and logical))	1-5 Likert scale
Rate the following elements/aspects in selected or preferred medical textbooks: (1 = poor, 5 = excellent) (language complexity (using words that I do not know their meaning))	1-5 Likert scale
Rate the following elements/aspects in selected or preferred medical textbooks: (1 = poor, 5 = excellent) (chapter summaries)	1-5 Likert scale
Rate the following elements/aspects in selected or preferred medical textbooks: (1 = poor, 5 = excellent) (end of chapter practice questions)	1-5 Likert scale
Rate the effectiveness of the following tools used in preferred or selected medical textbooks: (1 = least effective, 5 = most effective) (graphic elements (e.g., oxygen dissociation curve))	1-5 Likert scale
Rate the effectiveness of the following tools used in preferred or selected medical textbooks: (1 = least effective, 5 = most effective) (diagrams (e.g., DNA structure))	1-5 Likert scale
Rate the effectiveness of the following tools used in preferred or selected medical textbooks: (1 = least effective, 5 = most effective) (tables)	1-5 Likert scale
Rate the effectiveness of the following tools used in preferred or selected medical textbooks: (1 = least effective, 5 = most effective) (flowcharts)	1-5 Likert scale
Rate the effectiveness of the following tools used in preferred or selected medical textbooks: (1 = least effective, 5 = most effective) (mind maps)	1-5 Likert scale
Rate the effectiveness of the following tools used in preferred or selected medical textbooks: (1 = least effective, 5 = most effective) (sidebar linking different topics)	1-5 Likert scale
Rate the effectiveness of the following tools used in preferred or selected medical textbooks: (1 = least effective, 5 = most effective) (links to online sources (e.g., animation and videos))	1-5 Likert scale
Rate the effectiveness of the following tools used in preferred or selected medical textbooks: (1 = least effective, 5 = most effective) (clinical integration (clinical correlations to the basic concepts))	1-5 Likert scale
Rate the effectiveness of the following tools used in preferred or selected medical textbooks: (1 = least effective, 5 = most effective) (multidisciplinary integration (e.g., correlation between physiology and biochemistry))	1-5 Likert scale
Rate the following statements regarding preferred or selected medical textbooks: (increases my personal satisfaction for increasing my general medical knowledge)	Strongly agree/agree/neutral/disagree/strongly disagree
Rate the following statements regarding preferred or selected medical textbooks: (enables me to perform better in examinations)	Strongly agree/agree/neutral/disagree/strongly disagree
Rate the following statements regarding preferred or selected medical textbooks: (I found the information I was looking for)	Strongly agree/agree/neutral/disagree/strongly disagree
Which textbook format do you prefer most?	Paragraph/bullet point/other
Which of the following anatomy books, if any, do you use?	Text
Which of the following physiology books, if any, do you use?	Text
Which of the following histology books, if any, do you use)	Text
Which of the following embryology books do you use?	Text
Which of the following biochemistry books do you use?	Text
Which of the following genetics books, if any, do you use?	Text
Which of the following pathology books, if any, do you use?	Text
Which of the following immunology books, if any, do you use?	Text
Which of the following microbiology books, if any, do you use?	Text
Which of the following pharmacology books, if any, do you use?	Text
Which of the following clinical skills textbooks, if any, do you use?	Text
Which of the following internal medicine textbooks, if any, do you use?	Text
Which of the following general surgery textbooks, if any, do you use?	Text
Which of the following pediatrics textbooks, if any, do you use?	Text

## References

[1] Peterson MW, Rowat J, Kreiter C, Mandel J (2004). Medical students' use of information resources: is the digital age dawning?. Acad Med.

[2] Prasannan L, Gabbur N, Haughton M (2014). Use of web resources among medical students at a large urban medical center. Obstet Gynecol.

[3] Leff B, Harper GM (2006). The reading habits of medicine clerks at one medical school: frequency, usefulness, and difficulties. Acad Med.

[4] Carr NG (2010). The shallows: how the internet is changing the way we think, read and remember. https://www.nicholascarr.com/?page_id=16.

[5] Ellaway R, Masters K (2008). AMEE guide 32: e-learning in medical education part 1: learning, teaching and assessment. Med Teach.

[6] Wallace S, Clark M, White J (2012). 'It's on my iPhone': attitudes to the use of mobile computing devices in medical education, a mixed-methods study. BMJ Open.

[7] Davies BS, Rafique J, Vincent TR, Fairclough J, Packer MH, Vincent R, Haq I (2012). Mobile medical education (MoMEd) - how mobile information resources contribute to learning for undergraduate clinical students - a mixed methods study. BMC Med Educ.

[8] Baker LM, Gollop CJ (2004). Medical textbooks: can lay people read and understand them?. https://www.ideals.illinois.edu/items/1824.

[9] Wilson FL, Baker LM, Brown-Syed C, Gollop C (2000). An analysis of the readability and cultural sensitivity of information on the National Cancer Institute's Web site: CancerNet. Oncol Nurs Forum.

[10] Wilson FL, Mood DW, Risk J, Kershaw T (2003). Evaluation of education materials using Orem's self-care deficit theory. Nurs Sci Q.

[11] Gaitsgory O, Burgess A, Mellis C (2013). Opinion piece: 'medical students - learning from textbooks or electronic media?'. J Paediatr Child Health.

[12] Abrahamson S (1996). Essays on medical education.

[13] Brynhildsen J, Dahle LO, Behrbohm Fallsberg M, Rundquist I, Hammar M (2002). Attitudes among students and teachers on vertical integration between clinical medicine and basic science within a problem-based undergraduate medical curriculum. Med Teach.

[14] Dahle LO, Brynhildsen J, Behrbohm Fallsberg M, Rundquist I, Hammar M (2002). Pros and cons of vertical integration between clinical medicine and basic science within a problem-based undergraduate medical curriculum: examples and experiences from Linköping, Sweden. Med Teach.

[15] Elam CL, Wilson HD, Wilson EA, Schwartz R (1995). Physicians for the 21st century: implications for medical practice, undergraduate preparation, and medical education. J Ky Med Assoc.

[16] Geffen LB, Birkett DJ, Alpers JH (1991). The Flinders experiment in medical education revisited. Med J Aust.

[17] Ginzberg E (1993). The reform of medical education: an outsider's reflections. Acad Med.

[18] Morrison S, Cuneo R, Wainwright D, Stitz R (1993). Do the teaching hospitals of the University of Queensland really want a four-year medical course? A guarded yes. Med J Aust.

[19] Noronha Filho G, Resende JB, Lemme AC, Ney Júnior G, Frossard A (1995). Medical education and the integration of teaching and health care activities (Article in Portuguese). Rev Saude Publica.

[20] Richardson A (2002). An ecology of learning and the role of eLearning in the learning environment. https://www.semanticscholar.org/paper/AN-ECOLOGY-OF-LEARNING-AND-THE-ROLE-OF-eLEARNING-IN-Richardson/4614d56e37aec56570756e085ae43933f241f0e0.

[21] Collins J (2004). Education techniques for lifelong learning: giving a PowerPoint presentation: the art of communicating effectively. Radiographics.

[22] Murphy RJ, Gray S, Straja SR, Bogert M (2004). Student learning preferences and teaching implications. J Dent Educ.

[23] Winn JM, Grantham VV (2005). Using personality type to improve clinical education effectiveness. J Nucl Med Technol.

[24] Fleming ND, Mills C (1992). Not another inventory, rather a catalyst for reflection. J Educ Dev.

[25] (2024). Learning Styles: the multimedia of the mind. Research report. https://eric.ed.gov/?id=ED451140.

[26] Nesbit JC, Adesope O (2006). Learning with concept and knowledge maps: a meta-analysis. Rev Educ Res.

[27] Lujan HL, DiCarlo SE (2006). First-year medical students prefer multiple learning styles. Adv Physiol Educ.

[28] Chenoweth JD, Price K (1997). Using information technology at East Tennessee State University. Campus-Wide Inf Syst.

[29] Wehrwein EA, Lujan HL, DiCarlo SE (2007). Gender differences in learning style preferences among undergraduate physiology students. Adv Physiol Educ.

[30] Fleming ND (1995). I'm different; not dumb: modes of presentation (V.A.R.K.) in the tertiary classroom. Proceedings of the 1995 Annual Conference of the Higher Education and Research Development Society of Australasia (HERDSA).

[31] Cogdill KW, Moore ME (1997). First-year medical students' information needs and resource selection: responses to a clinical scenario. Bull Med Libr Assoc.

[32] Somekh B, Davis N (1997). Using IT effectively in teaching and learning: studies in pre-service and in-service teacher education. https://www.routledge.com/Using-IT-Effectively-in-Teaching-and-Learning-Studies-in-Pre-Service-and/Davis-Somekh/p/book/9780415121323.

[33] Perera T, Ranasinghe P, Perera U, Perera S, Adikari M, Jayasinghe S, Constantine GR (2012). Knowledge of prescribed medication information among patients with limited English proficiency in Sri Lanka. BMC Res Notes.

[34] Smith CA, Hetzel S, Dalrymple P, Keselman A (2011). Beyond readability: investigating coherence of clinical text for consumers. J Med Internet Res.

